# Atomic force microscopy in vitro study of surface roughness and fractal character of a dental restoration composite after air-polishing

**DOI:** 10.1186/1475-925X-9-59

**Published:** 2010-10-12

**Authors:** Marco Salerno, Luca Giacomelli, Giacomo Derchi, Niranjan Patra, Alberto Diaspro

**Affiliations:** 1 Italian Institute of Technology, via Morego 30, I-16163 Bolzaneto (Genova), Italy; 2 Tirrenian Stomatologic Institute, via Aurelia 335, I-55041 Lido di Camaiore (Lucca), Italy; 3 University of Genova, viale Causa 13, I-16145 Genova, Italy

## Abstract

**Background:**

Surface roughness is the main factor determining bacterial adhesion, biofilm growth and plaque formation on the dental surfaces in vivo. Air-polishing of dental surfaces removes biofilm but can also damage the surface by increasing its roughness. The purpose of this study was to investigate the surface damage of different conditions of air-polishing performed in vitro on a recently introduced dental restorative composite.

**Methods:**

Abrasive powders of sodium bicarbonate and glycine, combined at different treatment times (5, 10 and 30 s) and distances (2 and 7 mm), have been tested. The resulting root mean square roughness of the surfaces has been measured by means of atomic force microscopy, and the data have been analyzed statistically to assess the significance. Additionally, a fractal analysis of the samples surfaces has been carried out.

**Results:**

The minimum surface roughening was obtained by air-polishing with glycine powder for 5 s, at either of the considered distances, which resulted in a mean roughness of ~300 nm on a 30 × 30 μm^2 ^surface area, whereas in the other cases it was in the range of 400-750 nm. Both untreated surfaces and surfaces treated with the maximum roughening conditions exhibited a fractal character, with comparable dimension in the 2.4-2.7 range, whereas this was not the case for the surfaces treated with the minimum roughening conditions.

**Conclusions:**

For the dental practitioner it is of interest to learn that use of glycine in air polishing generates the least surface roughening on the considered restorative material, and thus is expected to provide the lowest rate of bacterial biofilm growth and dental plaque formation. Furthermore, the least roughening behaviour identified has been correlated with the disappearance of the surface fractal character, which could represent an integrative method for screening the air polishing treatment efficacy.

## Background

Dental caries is the most widespread disease, since it affects about 95% of the world population at some point during their lives [1]. Caries follow bacterial plaque formation, which arises after the increase in surface area accessible for bacterial adhesion due to the surface roughness associated with defects or damage of the dental structures [2-5]. In fact, the predominant role of surface roughness for bacterial adhesion with respect to other cofactors such as surface energy has already been clarified in the literature [6].

Traditional hand instruments or oscillating scalers used to remove dental plaque usually cause a significant increase in roughness of the underlying dental surfaces [3,7] made of either pristine or restorative material, causing in turn a faster re-growth of plaque in the time period following the treatment. Therefore, air-polishing (AP) with simultaneously ejected water and pressurized air containing abrasive powders has been introduced in dental cleaning, and is now routinely applied [8-10]. Sodium bicarbonate powder is largely used for AP [10]. Recently, glycine powder has also been tested in several in vitro, ex vivo and in vivo studies, demonstrating a good clinical efficacy and low abrasive effect [7,9,11-14].

Despite being the least invasive technique for the dental surfaces, even AP may result in surface damage [3,15], when the working parameters of type of abrasive powder, spraying time and distance are not correctly set. To date, AP surface effects have been studied by means of laser scanners or profilometers. One recognized advantage of these techniques lies in their ability to allow large areas characterization containing both untreated and AP treated regions. This makes it possible to measure the resulting defect depth and the absolute loss of material, and thus evaluate the integrity of the dental structures [12]. However, laser scanners and profilometers do not permit high-resolution measurement of the surface roughness. In this work we have performed an in vitro analysis on the effect of AP on the surface of a commercial material used in dental restoration using atomic force microscopy (AFM), which allows for a high resolution, direct quantitative characterization of the surface roughness [16,17]. Firstly, the AP treatment conditions resulting in the lowest dental structure damage - i.e. surface roughening - have been identified. Secondly, the effect of the different AP treatment conditions on the possible fractal character of the surface roughness has been analyzed. Surface feature patterns exhibit a fractal character when they are self-affine, meaning that similar patterns can be found when zooming in or out to different orders of magnitude of the lateral field of view. The fractal analysis has already been applied to dental surfaces for classification of dental patterns of different species in zoology [18] and for characterization of the wear patterns of bruxism [19], but to our knowledge has never been used in the analysis of AP of dental composite surfaces. This mathematical tool can provide a new way to account for the complexity of the topographical pattern of the treated material surface, which can in turn depend on the AP conditions. In fact, it is generally accepted that the measures of roughness from the distribution of heights z alone without any information on their spatial localization on the (x, y) plane is insufficient to completely describe the surface roughness [16].

## Methods

### Material used

As the dental reconstruction material to be characterized, a polymer composite recently made commercially available has been selected, namely Venus Diamond™, which is innovative in both organic matrix formulation and filler particles (with diameter in the wide range of 5 nm - 20 μm). Exact composition and other technical specifications of the material are proprietary to the manufacturer, (Heraeus Kulzer, Dübendorf, Germany).

### Specimen preparation

Slabs of the selected material were prepared in ambient air, by placing the material in rectangular plastic hollows used as a mold (5 mm × 5 mm lateral dimensions and 2 mm depth) and covering them with acetate strips. Exceeding material was pushed away by applying pressure over the strips with a quartz slide. The polymer composite was cured by irradiation for 40 s through the quartz slide with a blue LED lamp (Starlight Pro, Mectron, ITA), with total 5 W irradiation power across the emission spectrum (440-480 nm wavelength).

### Air-polishing

AP was performed using a standard commercial unit (Air Flow Handy 2, EMS SA, Nyon, Switzerland), with service air pressure of 3.5-4 bar (i.e. 51-58 psi) and instrument nozzle perpendicular to the specimen surface. Spraying distance was kept constant by means of a holding post, while spraying time was set via electronic control of an aperture. The instrument powder chamber was refilled after each AP run, to ensure maximum reproducibility of the powder jet.

The cured composite slabs were subjected to AP with either sodium bicarbonate powder or glycine powder (Air Flow Air and Air Flow Subgingival Perio, both from EMS SA, Switzerland). All possible combinations of different AP application times (t = 5, 10 or 30 s), and distances (d = 2 or 7 mm), were tested. These times and distances were chosen according to a previous study [11], after rescaling the time period to the treated surface area, which was kept constant to ~25 mm^2^, in order to obtain comparable treatment doses (in terms of time over unit area). Overall, 8 slabs (4 treated with bicarbonate and 4 treated with glycine) were prepared for each combination of time and distance, and 4 untreated slabs were used as the reference controls.

### Atomic force microscopy

The relative height maps of the sample surfaces, both for controls and AP treated specimens, have been acquired in air with a commercial AFM instrument (MFP-3D, Asylum Research, USA) operating in tapping mode. The used probes (NSG10, NT-MDT, Russia) had nominal spring constant and resonance frequency values of ~10 N/m and ~250 kHz, respectively. Optimum scan size was estimated to be S = 30 μm (i.e. scan area 30 × 30 μm^2^), chosen on the basis of the dimension of the typical bacteria that are expected to adhere to the dental composite surface in vivo [20]. The surface roughness of each specimen has been evaluated as the root mean square (RMS) value R_q _of the distribution of heights in the AFM topographical images.

### Statistical analysis of the roughness

The R_q _values have been analyzed with descriptive statistics. Comparison between different combinations of times and distances, and between powders, have been performed by means of ANOVA procedure with Bonferroni's post-hoc test, using SPSS software (SPSS 14, SPSS Inc, USA). A significance p value < 0.05 was considered to be statistically relevant.

### Fractal analysis of the texture

Generally, the R_q _value of surfaces with fractal profile can scale with both the image linear size S and the imaging time instant t, i.e. R_q _= R_q_(S,t) [21]. Dependence on S appears due to the limited field of view on the macroscopically wide surface. Dependence on t appears when the surface is associated with a physically evolving phenomenon, such as material deposition [22]. The general form of R_q_(S,t) is the Family-Vicsek scaling law [23] R_q _= t^β^·f(S/t^β/α^), where f, called scaling function, includes all the dependency on S, and α and β are the roughness and growth exponent, respectively. f should saturate to a constant for very high S values, such that R_q_~t^β^. For low S values, in turn, it should be f~S^α ^[23,24]. In our context we have investigated only the dependence of roughness R_q _on the AFM scan size S. We aimed to determine the R_q_(S) relationship for three different limiting cases, namely untreated samples, and samples having the surface treated with AP in a way typically resulting in the lowest and highest R_q_, that means 'least AP damaged' and 'most AP damaged' surfaces, respectively. For each specimen, the S in the imaging sequence has been increased in a 3× geometric ratio, from a minimum of 370 nm to the maximum allowed value of 90 μm (instrumental limit), for a total of 6 data-points.

## Results and discussion

### Considerations on the spatial resolution

In the AFM approach, while the best attainable resolution can reach the molecular scale, the main issue is the trade-off between resolution and scope, the latter being the scan size S. In fact, differing from optical and electron microscopy, the AFM images are digital maps where the measured quantity (cantilever deflection or oscillation amplitude) is serially sampled point by point at discrete spatial positions. Since the scan speed is relatively low due to the feedback response time (typically between 4 and 40 μm/s), a limited number of data-points is set, to maintain the overall image acquisition time to acceptable values (typically 2 to 20 min). Therefore, setting a given S value means setting the lower limit of achievable resolution to the value of S/√N, where N is the number of acquired data-points (i.e. image pixels). Details smaller than the pixel linear size S/√N are low-passed in the spatial frequency domain, and averaged out into the value measured at the considered position. In our case, with S = 30 μm and √N = 512, the smallest roughness features considered had linear size ~60 nm.

**Figure 1:**
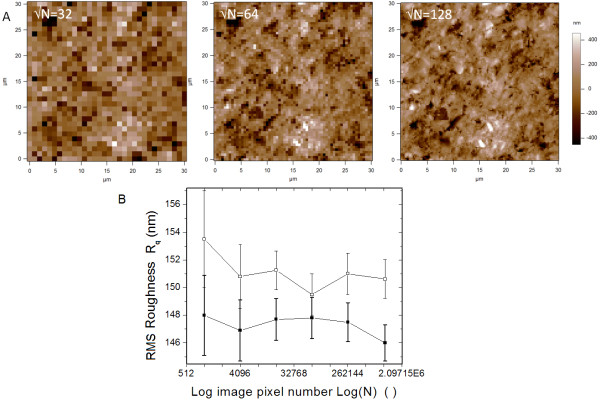
**Effect of pixel number of AFM images on the measured roughness**. A) AFM height images of a control specimen (untreated composite surface) for which the same region has been imaged (scan size S = 30 μm) with increasing number of pixels, N = 32^2^, 64^2^, and 128^2 ^from left to right. Height range is 900 nm for all images. Images not shown here completed the sequence, with N = 256^2^, 512^2^, and 1024^2^. B) Values or RMS roughness R_q _are reported for the whole set of AFM images with different N. Error bars represent semidispersion of forward and backward scans. Empty squares are from raw AFM data, filled squares are R_q _values after zero order image flattening. Lines are just guides to the eye.

In order to verify whether this limit may affect our measurements, we have preliminarily analyzed control samples by repeating AFM imaging in the same region with √N increasing from 32 to 1024, in a geometrical ratio 2×, for a total of 6 data-points. This process has been repeated three times in different regions. The obtained sequences of R_q _have all shown similar behavior. A representative case is reported in Fig. 1A, where the initial images (with lowest √N = 32, 64, and 128) of one such sequence are shown, which better describe the effect of insufficient and increasingly improved sampling of the surface features. In Fig. 1B the respective R_q _values of the whole sequence have been plotted versus the actual number of sampling points N. As N is rapidly increasing on doubling √N, a logarithmic scale (with base 10) has been used for the corresponding axis.

Two traces have been plotted in Fig. 1B, with R_q _values measured both from the AFM raw data (empty squares) and from the same data after zero order line-by-line flattening of the images (filled squares). The latter treatment is usually performed on the AFM images to remove artificial inconsistency among the different lines along the fast scan direction, due to drifts of the height offset in the instrument, which appears along the slow scan axis (vertical direction in our images). In this processing step a small amount of real R_q _can also be removed. Therefore, the 'true' R_q _value should lie between the two traces in Fig. 1B. In any case, the difference observed between the two traces is below 3%, with lower values for the flattened images (filled squares), as expected.

The error bars included in Fig. 1B are related to the difference between forward and backward scans of the same surface area. Despite the relatively large errors, lines have been traced that join consecutive data-points, which serve as guides to the eye. For both flattened and unflattened image data, these lines show a similar, roughly flat trend with Log(N), and comparable R_q _values but for the highest N points, for which the error bars of flattened and unflattened value do not overlap. Concerning the error bars, they appear larger for the two leftmost data-points of both plots in Fig. 1B, (i.e. for the two lowest N values). Indeed, when the real surface is properly sampled, the fluctuations are expected to decrease both in spatial frequency (i.e. N spacing) and amplitude (i.e. R_q _value and the respective standard deviation around its mean). In Fig. 1B both the mean R_q _values of flattened and unflattened images, their difference, difference, and the respective standard deviations (error bar lengths) reach a minimum at N = 256^2^.(fourth data-point starting from the left end of the plots). Therefore, our choice of N = 512^2 ^for all the AFM images in the subsequent analysis guarantees that no R_q _information from the analyzed surfaces is lost.

### Change in roughness upon air-polishing

**Figure 2:**
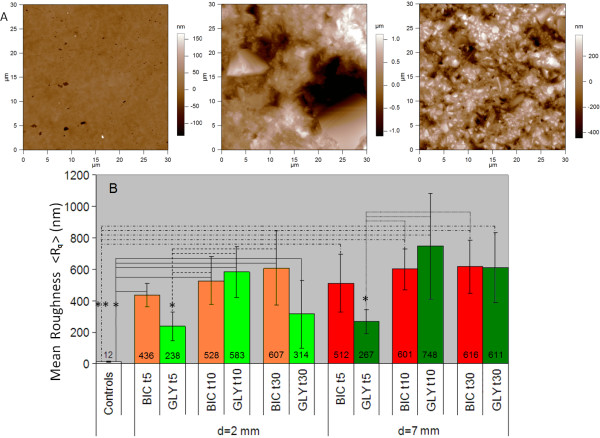
**Effect of different conditions of AP on the considered dental material**. A) Representative AFM height images with scan size S = 30 μm and pixel number N = 512^2 ^after different sample surface treatments. From left to right: control (untreated) specimen (height range 300 nm), specimen after AP for 5 s at a distance of 2 mm from the surface with bicarbonate (height range 2200 nm), and specimen after AP with the same time and distance but with glycine powder (height range 800 nm). B) Mean surface roughness <R_q_> of all samples, both controls and treated with AP at different combinations of powder (sodium bicarbonate, BIC, in red, or glycine, GLY, in green), time, and distance. Each value for a single sample is the mean of n = 8 values measured by AFM. Left half (light colors): time evolution of R_q _for both abrasive powders at a spraying distance of 2 mm from specimen surface; right half (dark colors): time evolution of R_q _for both abrasive powders at a spraying distance of 7 mm from specimen surface. * p < 0.05, ** p < 0.01.

Some AFM images of representative specimens are reported in Fig. 2A. The left panel in Fig. 2A shows the typical surface of a control specimen, whereas the middle and right panel show the surfaces of the composite material after AP at d = 2 mm and t = 5 s with bicarbonate and glycine powders, respectively. It may be qualitatively observed that the control is smoother than both treated specimens. Moreover, the specimen treated with glycine is smoother than the specimen treated with bicarbonate. In particular, sodium bicarbonate determined large depressions on the surface (typically 5-10 μm wide), whereas glycine was associated to smaller surface defects (typically 1-2 μm wide). These observations were consistent throughout most combinations of treatment distance and time.

Quantitative analysis of R_q _values confirmed these findings. The R_q _values resulting from the AFM images after AP for different t are represented in the Fig. 2B. The left half (light color bars) and the right half (dark color bars) of Fig. 2B refer to d = 2 and 7 mm, respectively.

For sodium bicarbonate (light and dark red bars) a trend towards an increase in R_q _over t can be observed; on the other hand, for glycine (light and dark green bars) the R_q _value reaches a maximum for the intermediate time t = 10 s, after which it seems to either decrease (for d = 2 mm, left half of Fig. 2B) or remain constant (d = 7 mm, right half of Fig. 2B). Overall, for both d = 2 and 7 mm, R_q _increased in all groups with respect to the controls; this effect was already evident after only t = 5 s treatment.

The difference in R_q _between treated specimens and controls was significant at all times for both powders (p < 0.01 for bicarbonate at all times and glycine for 10 s, p < 0.05 for glycine for 30 s), with the exception of glycine sprayed for t = 5 s. The application of glycine for t = 5 s was associated to the lowest R_q _value among all the treated samples, reaching a significant difference in most comparisons (p < 0.05 vs bicarbonate at all times and vs glycine for t = 10 s).

Even if a trend towards an increase of surface damage with the increase of d was observed as in previous studies [12,14], this difference was only significant for glycine sprayed for t = 30 s (p < 0.05). This can be partly due to the adjustment of the jet aperture cone at different spraying distance d, which was made to keep the treated area constant.

Overall, we have confirmed on a composite material used for dental restoration the observation - previously made in the literature only for natural teeth surfaces [11] - that during the AP process glycine powder determines less surface erosion than bicarbonate. Two different patterns for bicarbonate and glycine in the variation of R_q _over the treatment time have also been identified in our measurement. In principle, and according to a previous study [14], an increase in surface damage may be expected over time, at constant distance. This effect has been observed for bicarbonate powder, at both considered distances, but not for glycine. In fact, particularly at a spraying distance d = 2 mm a maximum of damage after AP for t = 10 s has been observed with this powder. Such an effect may be attributed either to a loss in power of the AP device over time when using glycine powder [14], which was not observed during the experimental process, or to the lower particle size of glycine. Indeed, glycine particles are about four times smaller than sodium bicarbonate particles [11]. On the basis of visual assessment by AFM, we may speculate that the larger bicarbonate particles remove larger portions of composite surface, thus resulting in a linear increase of R_q _at the adopted scan size S. On the other hand, glycine may determine smaller but most diffuse surface defects, determining a faster kinetics of damage, that may give rise to full surface coverage of defects, and thus result in a smoothing effect after removal of a whole material layer, within the considered treatment time (t = 30 s).

Concerning the clinical relevance of our measurements, comparison with the existing literature suggests that the RMS values reported in Fig 2 probably span the roughness range across which bacterial growth may indeed be activated or not, this step being found typically between ~200 nm and 800-2000 nm [24].

### Fractal character of the surfaces

Material surface features usually exhibit a fractal character right after growth [24,25], (for example, thermal evaporated metal films normally evolve in clusters with cauliflower-like structure, which is a typical form of fractal geometry [24,26]). Alternatively, a fractal character can arise as a consequence of surface treatment by physical or chemical methods [20,27-30]. In the present case, both conditions of as deposited material (after preparation of the composite slab) and material that has undergone a surface treatment (namely AP) appear as the candidates for occurrence of a fractal character.

The goal of the fractal analysis presented here was to search for a possible correlation between AP results and an additional roughness parameter other than simple R_q_. In the future, after proper in vivo testing of the treated surfaces, this novel measurement will possibly be checked against the clinical results of the obtained surfaces, such as the rate of bacterial growth.

In a Log-Log scale plot of R_q_(S) for a fractal surface, it is possible to identify α (roughness exponent) as the slope (see subsection "Fractal analysis of the surface"), which for the function R_q_(S) can be identified with the Hurst exponent H [21,24]. This coefficient can provide the fractal dimension D of the surface, if fractal in character, since it is D = D_E_-H, with D_E _dimension of the Euclidean space in which the considered object is embedded [21,25]. In our case it is D_E _= 3, as the AFM height images are 3D surfaces z(x, y).

**Figure 3:**
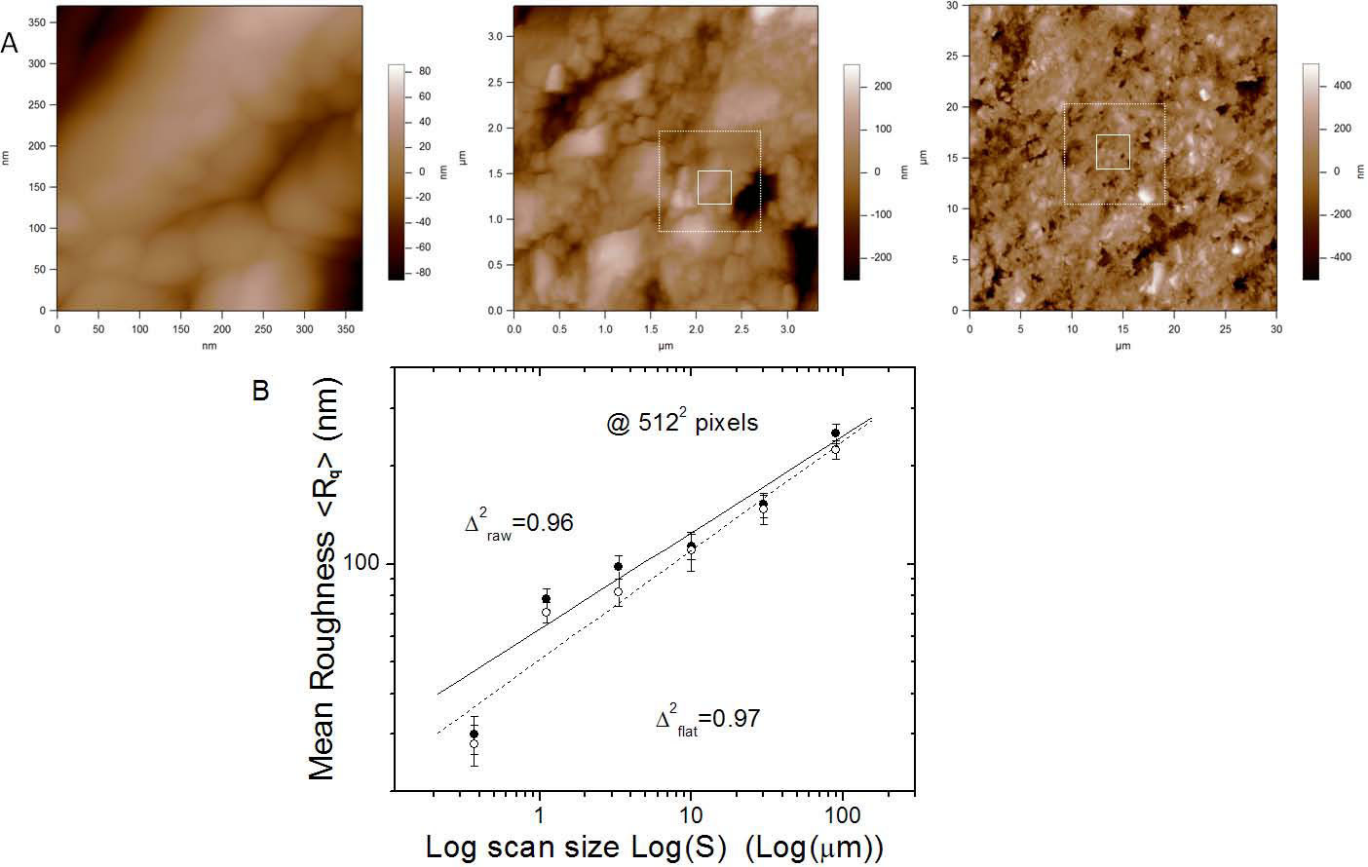
**Measurement of the fractal character from the AFM images**. A) Representative AFM height images with N = 512^2 ^pixels and increasing scan size S, from 0.37 to 3.33 on to 30 μm, from left to right. Height range is 180, 500 and 900 nm, respectively. Images not shown here completed the sequence, with S = 1.11, 10, and 90 μm. The white squares in the middle and right images represent the areas of previously imaged specimen regions. Two squares are traced since an image with intermediate S value had also been acquired between two consecutive shown images (S zoom factor: 3× at each step). B) Values of RMS roughness R_q _reported for one whole set of AFM images with different scan size S. Error bars represent semidispersion of forward and backward scans. Empty circles are from raw AFM data, filled circles are R_q _values after zero order image flattening. Lines are just guides to the eye.

As an illustrative example of our measurements, a subset of a sequence of images with increasing S has been included in Fig. 3A. For each sample, two specimens out of 6 were chosen, and for each specimen two different regions were imaged. The data-points in the plot are the mean of all the measurements at a given scan size, and the error bars represent ±1σ (standard deviation) ranges around them. Similar to the preliminary analysis of the effect of image resolution on R_q _(i.e. Fig. 1B), in all cases two curves, for raw data and for flattened images, have been plot.

In Fig. 3B the R_q _values for the control (untreated) specimens are reported with respect to S, in a Log-Log plot, (log base 10). Clearly R_q _increases over the whole S range considered, without reaching a plateau. With some deviation for the lowest S data-point, the measurements can be well fit by a straight line, with a common slope over more than three orders of magnitude for S. Therefore the surface displays a space-invariant relationship of its topographical features as they reflect in the R_q_(S) function, and appears to be fractal.

As the slopes from the fits in Fig. 3B are in the range 0.31 ± 0.02, the fractal dimension is D_control _= 2.69 ± 0.02.

**Figure 4:**
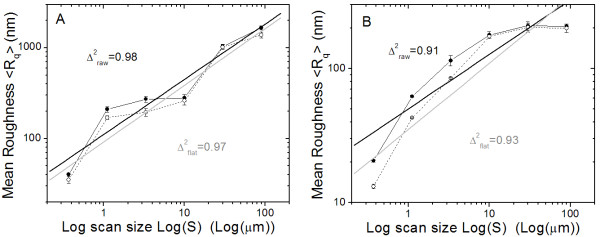
**Fractal character of AP treated samples**. A) and B) Similar plots as in Fig. 3B), for 'most AP damaged' (bicarbonate powder, d = 7 mm, t = 10 s) and 'least AP damaged' (glycine powder, d = 2 mm, t = 5 s) AP treatment conditions, respectively. Both raw (filled circles) and flattened (void circles) AFM data have been used. Straight thick lines are the linear regression curves for raw (black) and flattened (gray) data, respectively. Δ^2 ^numbers represent the residual deviations squared for the fit.

Similar sequences of AFM images have been acquired also for selected cases of AP treated samples, and the respective processing has been performed. In order to find possible differences associated with the AP conditions, samples with the most different resulting R_q _have been selected, according to statistical analysis. The 'least AP damaged' (i.e. lowest R_q_) sample was the set of specimens treated with glycine at d = 2 mm and t = 5 s, whereas for the 'most AP damaged' (i.e. highest R_q_) sample the specimens treated with bicarbonate at d = 7 mm and t = 10 s were selected. Same as for the control sample, the measurement has been repeated on two specimens from each sample, and on two different regions for each specimen. For each of the above four sets similar results were obtained, and in Fig. 4A and 4B representative sets for the 'most AP damaged' and for the 'least AP damaged' sample are reported, respectively. In these images the error bars only refer to the semidispersion of the forward and backward images on the same area.

It can be seen that the for the 'most AP damaged' sample set (Fig. 4A) the Log-Log R_q_(S) data-points followed also a roughly linear trend as for the control sample, which means that a fractal character is preserved throughout the respective AP treatment. In fact, the residual-square correlation coefficients Δ^2 ^between data-points and fitting lines in Fig. 4A is still as close to 1 as for the control data fits (see Fig. 3B). The slopes turn out to be 0.61 and 0.63 for the raw and for the flattened data in Fig. 4A, respectively, and 0.6 ± 0.1 for all the four sets altogether, such that the fractal dimension evaluated for this sample is D_mostDamage _= 2.4 ± 0.1. This is lower than the D_control_≅2.7 obtained for the control sample, possibly meaning that while still preserving the fractal character, the specimen surfaces treated in the considered AP conditions have undergone some loss of the complex structure arising from material deposition.

On the contrary, data-points in the 'least AP damaged' sample set (Fig. 4B) cannot be properly fit by a straight line. The slope values of the fitting straight line would be in the range of 0.45 ± 0.15 (for all the four sets altogether), corresponding to a D_leastDamage _= 2.55 ± 0.15, intermediate to D_mostDamage _and D_control _and compatible with both of them. However, the Δ^2 ^values of 0.91 and 0.93 show that no more fractal character of the surfaces appears over the whole S range considered, but this property has been removed by the optimized AP treatment.

In a previous work on fractal analysis of worn human dental surfaces [19], an increase in D appeared upon the decay of the surface quality, which was accompanied by an increase in R_q_. In our case, one could expect that conditions of minimum R_q _be associated with minimum D. In fact, the 'least AP damaged' sample cannot be compared with the 'most AP damaged' sample to this extent, as the former shows no fractal dimension at all. In turn, when comparing the control sample with the 'most AP damaged' sample a decrease in D, opposite to the increase in R_q_, appears; however, one should keep in mind that D_control _arises from material deposition, whereas D_mostDamage _arises from its later treatment, so they can be hardly correlated. Obviously AP destroys the former kind of fractal character, and, when not optimized, induces a new, generally not correlated fractal character.

## Conclusions

In this work we have determined in vitro the conditions for AP treatment of a given commercial composite for dental restoration (Venus Diamond) that, within a given set of combinations of working parameters (two abrasive powders of bicarbonate and glycine, two spraying distances of 2 and 7 mm, and three times of 5, 10, and 30 s) result in the lowest roughening of the composite surface. It is speculated that the same treatment applied to the same material in vivo should result in a reduced bacterial colonization rate. The best (i.e. least surface damage) AP conditions found are 5 s treatment with glycine powder at a distance of 2 mm. Glycine performed better than bicarbonate also at the other considered distance of 7 mm. Roughening resulting from AP at treatment times of 10 and 30 s was overall comparable for bicarbonate and glycine.

Three differently ranked AP conditions (untreated, 'least AP damaged', and 'most AP damaged') were further characterized by means of a fractal analysis of the spatial distribution of the surface roughness features. As a result, it was found that whereas the effect of the most damaging AP procedure is only a decrease in fractal dimension of the surface, the least damaging AP procedure destroys the correlation among the surface features, resulting in a disappearance of their fractal character. This finding suggests that the fractal analysis can be a helpful tool for a deeper characterization of dental surfaces.

The absence of experimental data on bacterial growth on our samples is one limitation of the present work. Such investigations are currently in progress in our laboratory, and will be the subject of a forthcoming report as soon as statistically relevant data will be available.

## Competing interests

The authors declare that they have no competing interests.

## Authors' contributions

LG conceived of the study, participated in its design, and performed the statistical analysis of Rq. GD carried out the specimen preparation and the AP treatment. MS participated in the design of the study and coordinated it, participated in the AFM measurements, performed the AFM image analysis to extract Rq values and fractal character, and drafted the manuscript. NP participated in the AFM measurements and helped draft the manuscript. AD revised the manuscript substantially.

All authors read and approved the final manuscript.
